# Determinants of Failure in the Reconstruction of the Tympanic Membrane: A Case-Control Study

**Published:** 2018-11

**Authors:** Francesco Dispenza, Alessia-Maria Battaglia, Pietro Salvago, Francesco Martines

**Affiliations:** 1 *Department of Otorhinolaryngology, A.O.U.P. “Paolo Giaccone”, Palermo, Italy.*; 2 *Department of Bio.Ne.C, University of Palermo, Palermo, Italy.*

**Keywords:** Chronic otitis, Ear, Myringoplasty, Middel ear, Surgery, Tympanoplasty

## Abstract

**Introduction:**

The recurrence rate after tympanoplasty is variable between 0% and 50%. The causes of failure may be different and frequently interrelated, making the surgical choice difficult and the prognosis not always favourable. In this study, we analysed recurrence rate and the possible causes of failure of tympanoplasty in the treatment of tympanic perforations.

**Materials and Methods:**

This prospective case-control study was carried out on patients undergoing tympanoplasty. The main outcome was closure of the tympanic membrane.

**Results:**

Among the studied 72 patients, the overall recurrence rate was 19.4%. The average follow-up was 28 months; no recurrence was observed over 12 months of follow-up. We observed a recurrence of 30.7% (OR 2.9) in near total perforations. In 32 subjects with a perforation of over half size of the membrane, a recurrence rate of 31.2% was noted (OR 4.09; P< 0.05). In 22 out of the 72 patients, there was a bilateral chronic otitis where the rate of recurrence was 27.2% (OR 1.9). During the postoperative period, 10 patients contracted infection of the middle/external ear, and in all of these cases failure of the surgical intervention was recorded (P<0.01).

**Conclusion:**

The rate of recurrence is closely related to several factors that may be concomitant and therefore, worsen the prognosis. Perforations that affect more than 50% of the tympanic surface are related to a higher rate of failure and are often associated with one of the two conditions previously described. Postoperative infection is the most significant risk factor for recurrence.

## Introduction

Tympanic membrane reconstruction is a relatively common surgical procedure in otorhinolaryngology. The modern concept of tympanic repair has been described by Berthold introducing the term myringoplasty when describing repair of the tympanum in 1878 (1). With the advent of the surgical microscope, the concept of tympanic reconstruction and, consequently, the surgical techniques changed and in 1951; Wullstein and Zollner introduced tympanoplasty in their famous classification(2). Tympanoplasty with its "new" feature was applied in the reconstruction of the tympanic membrane with the possibility of ossicular continuity restoration. Over time, the terms myringoplasty and type I tympanoplasty were used interchangeably causing some terminological confusion; in fact, tympanoplasty is used for those procedures that require elevation of the tympanum-meatal flap, unlike myringoplasty (3). The success of surgery is obviously linked to the anatomical and, possibly, functional restoration of the tympanic membrane. In the literature, the recurrence rate is variable with widely heterogeneous data and ranges between 50% and 100% (4). The causes of failure may be different and frequently interrelated, making the surgical choice tentatively indicated and the prognosis not always favourable(5).

In this case-control study, we aimed to analyse the rate of recurrence of tympanic perforation after tympanoplasty and the possible causes of failure, that is, factors related to perforation features, factors related to ear clinical conditions, and factors related to surgical technique.

## Materials and Methods

This case-control study was designed to prospectively evaluate patients with chronic otitis media with tympanic perforation during 2014-2016. We included patients eligible for tympanoplasty, which involves elevation of a tympanomeatal flap and access to the middle ear. The exclusion criteria consisted of patients with clinical or intraoperative evidence of cholesteatoma and paediatric patients (under 16 years old). All the patients underwent micro-otoscopy and video-otoscopy with recording of images, nasal and nasopharyngeal endoscopy to visualize the Eustachian tube opening, pure tone audiometry (0,5,1,2,4 kHz), and speech audiometry and tympanometry of the healthy ear in those cases with unilateral perforation.

The primary post-surgical endpoint was considered complete closure of the tympanic membrane defined as intact at least 12 months after the surgery. The secondary endpoints were considered: adverse events (i.e., anterior blunting, recurrent perforation, tympanic lateralization, and iatrogenic cholesteatoma) and improvement of the auditory threshold (closure of the bone-to-air gap). The variables considered were patient age, follow-up duration, perforation size (considering 25% as one quadrant by dividing the whole tympanic membrane into four quadrants in video-otoscopic image), perforation site, contralateral ear condition (i.e., normal and chronic otitis media), middle ear conditions (i.e., ear drainage and mucosa features), underlay or overlay tympanoplasty technique regarding the placement of the graft with respect to the remnants of tympanic membrane, cartilage graft use, calibration of the external bony auditory canal, and anterior anchorage of the graft by a small fascial flap under the anterior annulus. All the procedures were performed by the same surgeon (FD) to exclude operator bias and the surgical technique in all the patients was autologous graft of temporal fascia. 

The analysis of risk factors with Odds Ratio and Chi-squared test was performed using MS Excel software, considering as case those patients with recurrence of tympanic membrane perforation and control those with complete restoration.The size of the study was considered enough after two years of observation of the patients meeting the inclusion criteria. All the patients provided an informed consent to the surgical procedure and our institutional review board approved this study.

## Results

Seventy-two patients matched the inclusion criteria and were analysed (28 M and 44 F; mean age 39.4 years). Overall, cases of recurrence after the first intervention were 14 (19.4%). Only eight patients needed surgical revision, of which two relapsed again, but these subsequent procedures were not included in this analysis. The minimum follow-up was 14 months to a maximum of three years with an average of 28 months; recurrence within one month from the surgical intervention was observed in 10 out of 14 patients and 4 within 3-6 months. Age was not significantly associated with recurrence (P>0.05); patients were subdivided into three groups (Table.1) of 16-30 years old with a recurrence rate of 15.4% (OR 0.65), 31-50 years old with a recurrence rate of 15.4% (OR 0.65), and over 50 years old with a recurrence rate of 30% (OR 2.35). Three groups were identified regarding the anatomical site of perforation, namely 14 patients with anterior perforation, 32 with posterior perforation, and 26 with near total perforation extended to both anterior and posterior quadrants; no recurrence was recorded for the first group, while we had a recurrence of 18.7% in the posterior perforation and 30.7% (OR 2.9) in near total perforation groups. By evaluating the size of the perforation regardless of the site, we noted a significant difference between near-total and small perforations (P< 0.05). 

**Table 1:** Risk factors for recurrence in tympanoplasty. The percentage reported in bold characters had an Odds Ratio > 2.0; the P-value reported in bold had statistical significance.

		**Recurrence**				**Hearing Improvement**	
		**Yes**	**No**	**%**	**p-value**	**Yes**	**No**
Age	16-30	4	22	15.4	> 0.05	20	6
	31-50	4	22	15.4		20	6
	> 50	6	14	30		15	5
Site of perforation	Anterior	0	14	0	> 0.05	13	1
	Posterior	6	26	18.7		25	7
	Neartotal	8	18	30.7		17	9
Size of perforation	< 50%	4	36	10	< 0.05	35	5
	> 50%	10	22	31.2		22	12
Tympanosclerosis	Yes	4	20	16.6	> 0.05	19	5
	No	10	38	20.8		36	12
Bilateral Chronic Otitis	Yes	6	16	27.2	> 0.05	15	7
	No	8	42	16		40	10
Retraction Pocket	Yes	0	14	0	> 0.05	14	0
	No	14	44	24.1		41	17
Preoperative Draining Ear	Yes	4	10	28.5	> 0.05	8	6
	No	10	48	17.2		47	11
Postoperative Infection	Yes	10	0	100	< 0.01	0	10
	No	4	58	6.4		55	7

Forty patients had a perforation that affected less than half of the tympanic surface, in this group a 10% recurrence rate was noted; the other 32 subjects had a perforation of over half the size of the membrane with a recurrence rate of 31.2% (OR 4.09). 

In 22 out of 72 patients (27.2%, OR 1.9), there was a bilateral chronic otitis media. Preoperative ear drainage was present in 14 patients with a recurrence rate of 28.5% (OR 1.9). 

**Fig 1 A,B:**
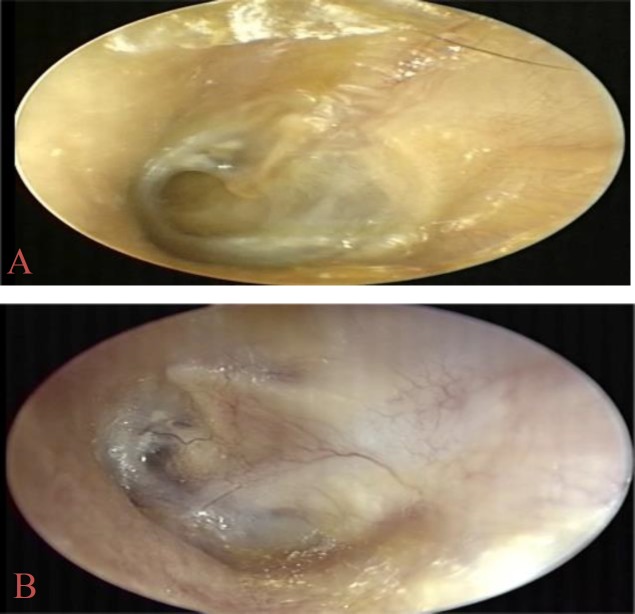
Tympanoplasty with temporalis fascia underlay graft. A: preoperative tympanic perforation in anterior site. B: postoperative otoscopy with perforation closure

**Fig 2 A, B:**
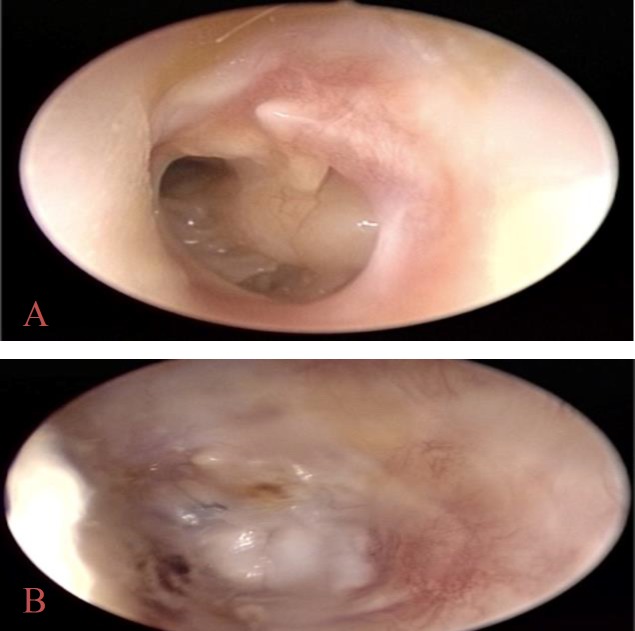
Tympanoplasty with cartilage and perichondrium overlay graft. A: preoperative tympanic near total perforation. B: postoperative otoscopy with perforation closure

For the reconstruction of the tympanum, the underlay technique was adopted in 67 patients (Fig.1 A,B) and in five patients the overlay technique was used (Fig.2 A,B) with a recurrence rate of approximately 20% in both groups (OR 0.9). 

We observed a 30.7% recurrence rate (OR 2.9) in patients with anterior anchoring, without statistical significance (P>0.05). Other findings related to surgical technique are shown in (Table.2). It is worth mentioning that not all significant differences are related to relapse or onset of complications. During the postoperative period, 10 patients had an infection of the middle/outer ear and in all cases a tympanic perforation with failure of the surgical intervention was recorded with statistical significance (P<0.01). Auditory improvement was achieved in 55 out of 72 patients (76.4%), and 17 patients maintained pre-operative hearing. No patients presented worsening in hearing threshold. Analysing the concomitance of several factors in patients with recurrence, postoperative infection was associated with perforation that involved over 50% size of the tympanic membrane.

**Table 2:** Recurrence rate analyzed by surgical technique parameters. In bold character percentage with Odds Ratio > 2.0

		**Recurrence**				**Hearing Improvement**	
		**Yes**	**No**	**%**	**p-value**	**Yes**	**No**
Surgical technique	Underlay	13	54	19.4	> 0.05	51	16
	Overlay	1	4	20		4	1
Canalplasty	Yes	8	36	18.1	> 0.05	34	10
	No	6	22	21.4		21	7
Anterior Anchorage	Yes	8	18	30.7	>0.05	17	9
	No	6	40	13		38	8
Cartilage Graft	Yes	0	6	0	>0.05	5	1
	No	14	52	20.5		50	16
Ossicular Chain Reconstruction	Yes	1	11	8.3	>0.05	11	1
	No	13	47	21.6		44	16
Revision Surgery	Yes	0	6	0	>0.05	4	2
	No	14	52	21.2		51	15

## Discussion

Multiple factors may affect the success rate of tympanoplasty and many of them are concomitant in the same patient making the surgical choice tentatively indicated and accompanied with variable risk of failure and recurrence of tympanic perforation (6,7). In our analysis, the first factor considered was the age of the patients, and we observed that in the over 50 years age group the rate of recurrence was higher than the average, setting the age as a negative prognostic factor in the failure of the procedure with moderate relative risk, but with low statistical significance.

Our results indicated that perforations affecting more than 50% of the tympanic surface have a relatively high risk of recurrence in line with literature (4,6). The anterior or posterior perforation site does not seem to affect the results; therefore, perforations involving multiple sites are inevitably linked to the same result as perforations affecting over 50% of the membrane with a recurrence rate of approximately 30%. The reasons why a large perforation has a lower success rate lie, probably, in technical difficulties that reduce visibility during reconstruction in a reduced overlap surface between graft and residual tympanum and diminished support for grafting resulting in reduced vascular supply(8,9). 

The conditions of the middle ear mucosa and the simultaneous presence of otorrhea in our cases appear to be a negative prognostic factor regarding recurrence with an augmented relative risk (OR 2.9). The literature has not defined a clear role for this variable in tympanoplasty outcomes and variable results have been reported in this regard (10-12).

Patients with bilateral chronic otitis had a recurrence rate almost twice as that in patients with unilateral pathology with a relative risk of 2.9, probably confirming the role of the Eustachian tube in the middle ear physiology and consequently, in anatomical integrity of the tympanic membrane(6,13-15). 

The surgical technique adopted has not been proven to be effective in outcomes, although the overlay technique was used in a limited number of cases making the statistical comparison not possible. The graft was positioned medially at the hammer handle with underlay or overlay technique, and in half of the cases a calibration of the external auditory canal with a drill was performed in order to better visualize the area to be reconstructed in case of narrow or tortuous auditory canal, also this parameters were unrelated to the risk of recurrence. 

Based on our data, it was initially found that anterior anchorage of the graft between the fibrous annulus and the bony annulus with an "eyelet" of about 2-3 mm appeared to be correlated with a higher rate of recurrence. In fact, this technique has been used in all cases of subtotal perforation to ensure better support for graft. Comparison of the rates of recurrence between patients with near total perforation and those with an anterior anchorage depicted that the rates of failure were similar, and the cause of recurrence is more likely to be related to the perforation size and not to the surgical technique.

As widely acknowledged in the literature, the use of cartilage, although only limited to six cases, proved to be a positive prognostic factor giving the graft an increased resistance (16).

A decisive factor in the success of tympanoplasty was the incidence of post-operative infections, despite oral antibiotic administration during the first post-operative week. In our patients, 10 cases of postoperative infection occurred within the first two weeks of the intervention and before the removal of the external auditory canal dressing. In all the cases, perforation recurrence was observed. Infection of the operated region was the main cause of recurrence in 10 patients out of 14; the remaining four patients had relapsed in a variable period of 3-6 months after surgery. Infection risk may be caused by controllable and uncontrollable factors. With a post-operative empirical antibiotic treatment administered over a week, it is possible to reduce the risk of infection. However, other random factors often linked to poor patient compliance with suggested post-operative indications (e.g., do not wet the ear and do not remove medications) can seriously compromise the post-operative course and cannot always be remedied with subsequent dressings.

The concomitance of preoperative ear drainage or bilateral chronic otitis, as well as large perforations and postoperative infection can result in failure of the surgical procedure, which indicates that greater attention should be paid to these patients. Therefore, planning a reconstruction with a more durable support and drying the ear as much as possible before the surgery seem to be necessary (17,18).

Generally, the follow-up duration does not seem to affect the anatomical success of tympanoplastyand our study showed that recurrence after 12 months is rare (or absent) (4,8,11). 

## Conclusion

The recurrence of tympanic perforation represents the main failure of tympanoplasty. The rate of recurrence is closely related to several factors that may be concomitant and therefore, deteriorate prognosis. With regard to surgical technique, it seems that all techniques have the same success rates. Chronic ear drainage and chronic bilateral otitis with tympanic perforation are two parameters that reduce the chance of success. Perforations that affect more than 50% of the tympanic surface are related to a higher rate of failure and are often associated with one of the two conditions previously described. Postoperative infections cause most of recurrences and may be the main unpredictable and uncontrollable cause, in particular, if linked to patient’s compliance with postoperative indications.
